# The Role of Sodium Glucose Co-Transporter 2 Inhibitors in Atrial Fibrillation: A Comprehensive Review

**DOI:** 10.3390/jcm13185408

**Published:** 2024-09-12

**Authors:** Panagiotis Stachteas, Athina Nasoufidou, Efstratios Karagiannidis, Dimitrios Patoulias, Paschalis Karakasis, Sophia Alexiou, Athanasios Samaras, Georgios Zormpas, George Stavropoulos, Dimitrios Tsalikakis, George Kassimis, Christodoulos Papadopoulos, Nikolaos Fragakis

**Affiliations:** 1 Second Cardiology Department, Medical School, Hippokration General Hospital, Aristotle University of Thessaloniki, 54124 Thessaloniki, Greece; staxteasp@gmail.com (P.S.); athinanassi@gmail.com (A.N.); stratoskarag@gmail.com (E.K.); pakar15@hotmail.com (P.K.); sophiealexiou@yahoo.com (S.A.); ath.samaras.as@gmail.com (A.S.); sparky.zorb@gmail.com (G.Z.); stavropoulosgeo@gmail.com (G.S.); gksup@yahoo.gr (G.K.); 2 Outpatient Department of Cardiometabolic Medicine, Second Cardiology Department, Medical School, Hippokration General Hospital, Aristotle University of Thessaloniki, 54124 Thessaloniki, Greece; dipatoulias@gmail.com; 3 Department of Electrical and Computer Engineering, University of Western Macedonia, 50100 Kozani, Greece; tsalikakis@gmail.com; 4 Third Cardiology Department, Medical School, Hippokration General Hospital, Aristotle University of Thessaloniki, 54124 Thessaloniki, Greece; chrpapado@gmail.com

**Keywords:** SGLT-2 inhibitors, gliflozins, atrial arrhythmias, AF incidence, AF recurrence, AF episodes, antiarrhythmic

## Abstract

Atrial fibrillation (AF) is the most prevalent arrhythmia among adults worldwide, frequently co-occurring with comorbidities such as Heart Failure (HF) and Type 2 Diabetes Mellitus (T2DM). This association contributes to increased morbidity and mortality, elevated healthcare costs, and diminished quality of life. Consequently, preventing or delaying the onset and recurrence of AF is crucial for reducing the incidence of complications. Sodium-glucose cotransporter 2 inhibitors (SGLT2is), due to their multifaceted pharmacological actions, have been proposed as potential therapeutic agents in the management of AF. However, current evidence from both animal models and clinical studies remains inconclusive. This narrative literature review aims to provide a comprehensive analysis of existing evidence on the impact of SGLT2is on the prevalence, incidence of new-onset, and recurrence of AF in diabetic populations and patients with HF. Numerous observational studies, predominantly retrospective, suggest a consistent reduction in AF risk with SGLT2is, while randomized controlled trials (RCTs) have yielded mixed results, with some demonstrating benefits and others not reaching statistical significance. The heterogeneity in study outcomes, population characteristics, follow-up duration, and specific SGLT2is used, as well as potential biases, underscore the need for further extensive and rigorous RCTs to establish definitive conclusions and elucidate the underlying mechanisms.

## 1. Introduction

Atrial fibrillation (AF) is the most prevalent arrhythmia among adults globally. Its prevalence in the general population is estimated to be between 2–4%, increasing with age to 5–10% in individuals aged 70 years and older [1]. Concurrently, in recent decades, the incidence of AF has shown a significant rise, likely due to the aging population and the increased frequency of diagnosis facilitated by advanced diagnostic tools [1]. The primary complication of AF is systemic embolism, particularly cerebrovascular events, resulting from thrombi that typically form within the left atrial appendage. With the aging population, it is projected that the prevalence of AF will increase from 1.9% in 2008 to 3.5% by 2050, and the number of AF-related stroke and systemic embolism incidents will triple between 2010 and 2060 in both developing and developed countries [2].

Additionally, AF is associated with an increased incidence of Heart Failure (HF), leading to higher morbidity and mortality, increased medical costs, and reduced quality of life [1,3], as indicated by recent observational studies [4,5]. AF is also linked to various diseases, such as Type 2 Diabetes Mellitus (T2DM), metabolic syndrome, chronic kidney disease (CKD), and coronary heart disease (CHD), as well as Major Adverse Cardiovascular Events (MACEs), highlighting its status as a significant modern public health issue [6,7].

Sodium-glucose cotransporter 2 inhibitors (SGLT2is) represent a relatively novel category of oral hypoglycemic agents that function by impeding the renal reabsorption of sodium and glucose. This action decreases the renal threshold for glucose absorption in the proximal tubule of the nephron, thereby inducing glycosuria and natriuresis [8]. Until now, five different SGLT2is (or gliflozins), namely, dapagliflozin, canagliflozin, empagliflozin, ertugliflozin, and bexagliflozin, have been approved by the US Food and Drug Administration (FDA) for the treatment of T2DM as an adjunct therapy to diet and exercise [9]. Initially, SGLT2is were employed as orally administered antidiabetic medications in patients with T2DM, offering additional cardiovascular (CV) benefits, including the regulation of blood pressure (BP) and body weight [8,10]. In clinical settings, SGLT2is are typically used to pharmacologically manage T2DM as a second- or third-line treatment when metformin and/or sulfonylureas fail to achieve adequate glycemic control, or as first-line agents for high-risk individuals with established CV diseases, HF, or CKD [11,12]. However, in recent years, systematic research has revealed their surprisingly significant benefit in HF patients across the spectrum of Left Ventricle Ejection Fraction (LVEF) irrespective of underlying diabetic status. Consequently, SGLT2is have been recommended for administration to HF patients with both reduced and preserved LVEF in the latest guidelines from both the American Heart Association [13] and the European Society of Cardiology [14], as they have shown an efficacy in decreasing HF hospitalizations and CV mortality, a benefit corroborated by recent meta-analyses [15,16], underscoring their crucial role in the treatment of HF.

Over the past decade, there has been significant clinical interest in preventing or delaying the onset and recurrence of AF due to the potential to markedly decrease the incidence of complications such as stroke and HF exacerbation. SGLT2is, owing to their multifaceted actions, have been proposed as valuable agents in the pharmacological management of AF through their direct and predominantly indirect antiarrhythmic properties [3,17]. Nonetheless, current evidence from both animal models and clinical research studies remains inconclusive [18]. Hypotheses have been formulated regarding the clinical benefits of SGLT2is in the prevention and reduction of AF episodes. However, the majority of published meta-analyses emphasize that the outcomes from the DECLARE-TIMI 58 trial (involving dapagliflozin) predominantly influence the overall conclusions concerning the prevention of atrial arrhythmias [19].

The objective of this narrative literature review is to deliver a thorough analysis of the current evidence concerning the impact of SGLT2is on the prevalence, incidence of new-onset, and recurrence of AF in both diabetic populations and patients with HF. The primary focus is on efficacy data derived from randomized clinical trials (RCTs) and observational studies that evaluate the effects of gliflozins on AF episodes. Additionally, this review explores the potential underlying pathological mechanisms involved in the antiarrhythmic properties of SGLT2is.

## 2. Pathophysiological Mechanisms

### 2.1. Mitochondrial Dysfunction and Oxidative Stress

One of the main pathophysiological mechanisms associated with the genesis of AF is the mitochondrial dysfunction of the atrial cells [20]. Normally, healthy cardiomyocytes use predominantly a mixed metabolism pattern utilizing both fatty acids and glycolytic products. However, in patients with T2DM, due to insulin resistance, a shift of metabolism towards fatty acids and less towards carbohydrates has been observed, a metabolic pathway which is less efficient in terms of ATP production and is associated with structural and functional changes (e.g. reduced oxidative phosphorylation) in atrial mitochondria, thus creating a arrhythmogenic substrate and contractile dysfunction [21]. Reactive oxygen species (ROS) derived from mitochondrial dysfunction cause significant anatomical and functional alterations in the atrial myocardium (fibrosis, hypertrophy, dilation) and have a proarrhythmic effect facilitating the genesis and progression of AF [22].

Emerging data from basic science and animal model studies demonstrate an interaction between SGLT2is and the atrial myocardium. Indeed, data from experimental animal studies have shown that SGLT2is can reduce oxidative stress and ameliorate mitochondrial respiratory capacity and intracellular calcium handling, thereby leading to reversal of myocardial structural and electrical remodeling [23,24,25]. In a recent study, SGLT2is demonstrated the ability to increase mitochondrial biogenesis partially due to metabolic alterations like ketone body formation [26], and empagliflozin was observed to potentially restore mitochondrial dysfunction [27]. In another related study, administration of empagliflozin to diabetic rats significantly prevented the development of atrial myopathy and improved atrial mitochondrial respiratory function and biogenesis acting as a preventive agent of oxidative stress and consequently DM-related AF [28]. In addition, canagliflozin demonstrated its favorable antiarrhythmic impact on AF in a canine model by mitigating intra-atrial fibrosis and improving electrophysiological parameters, thus diminishing AF inducibility [29].

A considerable body of evidence suggests that the characteristic response pattern elicited by SGLT2is can be attributed to their ability to enhance cellular autophagy, potentially through the simultaneous upregulation of nutrient deprivation pathways and downregulation of nutrient excess pathways. This is evidenced by increased expression and activity of key metabolic regulators, including AMPK (adenosine monophosphate-activated protein kinase), SIRT1, SIRT3, SIRT6 (sirtuins), and PGC1-α (peroxisome proliferator-activated receptor γ coactivator 1-α), alongside reduced activation of mTOR (mammalian target of rapamycin). The distinct cardioprotective and renoprotective effects of SGLT2is are nullified when autophagy, AMPK, or sirtuins are inhibited or knocked down [30].

### 2.2. Inflammatory Response and Atrial Fibrosis

Chronic inflammation and oxidative stress are well-established factors contributing to the development and progression of atrial anatomical and electrical remodeling, particularly in diabetic populations [22]. In patients with T2DM, atrial fibrosis—mediated and exacerbated by the increased production of advanced glycation end products and elevated expression of TGF-β—is a major trigger for the development of reentrant arrhythmias including AF [3]. Accumulating evidence from experimental and human studies suggests that SGLT2is could be beneficial to myocardial and endothelial tissue by attenuating the inflammatory response and the abnormal activation of fibroblasts [31,32]. These benefits are achieved through the reduction of inflammatory cell infiltration in cells [32,33,34], the suppression of circulating pro-fibrotic, pro-inflammatory and inflammatory cytokines such as CRP, TNF-α, IL-6, TGF-β, type I collagen, matrix metalloproteinase 2, and adipocytokines [32,33,35,36], the regulation of NO bioavailability [37], and the modulation of NLRP-3 inflammasome activity in human macrophages, leading to decreased release of IL-1β and IL-18 [38]. Additionally, epicardial adipose tissue (EAT) has been described as highly metabolically active, with atherogenic, profibrotic, proinflammatory, and arrhythmogenic properties [39,40,41], and it is closely related with the onset and progression of both CAD and AF [40,42,43,44,45]. Although only a few studies have examined the potential impact of SGLT2is on EAT, preliminary findings suggest that SGLT2is reduce EAT [46], thereby expanding their anti-inflammatory and antiarrhythmic benefits [47].

### 2.3. Uric Acid—Ion Balance—Diuretic and Natriuretic Effects—Diastolic Function

The potential pathophysiological mechanisms underlying the beneficial effects of SGLT2is on preventing the onset and progression of AF are multifaceted and extend beyond the enhancement of mitochondrial function, redox biology, and attenuation of chronic systemic inflammation. Hyperuricemia has been correlated with the incidence and recurrence of atrial arrhythmias [48], and SGLT2is may confer protective effects against the development of AF by reducing plasma uric acid levels [49,50]. Furthermore, low levels of plasma magnesium are associated with an increased incidence of AF due to its promotion of elevated sinus automaticity and supraventricular ectopy [51]. SGLT2is promote the reabsorption of Mg^2+^ at the nephron and so contribute to the maintenance of normal serum Mg^2+^ levels by improving insulin sensitivity, thereby preventing hypomagnesemia and its associated arrhythmogenic effects [52,53]. Nevertheless, potassium disturbances increase the risk of AF [54,55], while the use of SGLT2is, especially in populations vulnerable to potassium disturbances, including people with T2DM or HF, has been shown to reduce the risk of both hypo- and hyperkalemia [56].

In HF and T2DM patients, excess Na^+^ enters into myocardial cells mainly through the sodium-glucose cotransporter, the expression of which is usually upregulated, causing Na^+^ overload and consequently arrhythmogenic substrate [57,58]. SGLT2is may demonstrate their antiarrhythmic benefits via the attenuation of intracellular Na^+^ overload [17]. Also, SGLT2is inhibit late-I_Na_ in cardiomyocytes by binding to Nav1.5 sodium channels, exhibiting cardioprotective and antiarrhythmic properties [59]. Except for SGLT and late-I_Na_ inhibition, according to experimental studies, SGLT2is reduce intracellular Na+ by inhibiting sodium-hydrogen exchanger 1 (NHE-1), reversing calcium overload regardless of diabetes status [60]. Data from animal studies reveal that empagliflozin can modify Ca^2+^ regulation and Na^+^/hydrogen-exchanger currents, contributing to its antiarrhythmic properties [61]. Furthermore, empagliflozin reduces calmodulin-dependent kinase II (CaMKII) activity and CaMKII-dependent sarcoplasmic reticulum Ca^2+^ leak, which improves myocardial calcium homeostasis and helps alleviate contractile dysfunction and arrhythmias, offering additional benefits in patients with HF and T2DM [62]. Similarly, dapagliflozin contributes to inhibiting Ang II-induced ox-CaMKII upregulation, leading to beneficial modification of the electrical and structural atrial substrate [63].

In addition, the diuretic effect of SGLT2is may contribute beneficially in AF prevention. These inhibitors reduce Na^+^ reabsorption by inducing constriction of the proximal arteriole and dilation of the distal arteriole, thereby lowering the GFR [64]. Given that the majority of Na^+^ reabsorption occurs in the loop of Henle and the distal tubule, SGLT2is exert minimal diuretic effects on their own. However, in patients with HF, they can ameliorate the diuretic response when combined with other diuretic agents by improving responsiveness to atrial natriuretic peptide [64]. Furthermore, SGLT2is significantly promote natriuresis, facilitating the removal of excess fluid from the body [65]. By inhibiting the reabsorption of glucose and sodium in the proximal renal tubules, these agents increase the urinary excretion of both substances, resulting in a net reduction in extracellular fluid volume and, subsequently, cardiac preload, and finally in atrial volume [66]. Considering that the LV diastolic dysfunction including the atrial dilation and the stretching of cardiomyocytes in the pulmonary veins and atria due to increased filling pressures are primary instigators for the onset of AF [67], the improvement in LV diastolic function and BP reduction mediated by SGLT2is may alleviate these triggers and potentially prevent not only AF genesis and progression, but also the future development of HFpEF, especially among high-risk patients [68,69,70].

### 2.4. Hypoglycemia and Body Weight Reduction

Variability in blood glucose levels, particularly hypoglycemia, is associated with an increased incidence of AF in patients with T2DM [71]. SGLT2is lower glucose levels through mechanisms not involving pancreas and insulin secretion, primarily by reducing renal glucose reabsorption. This results in a minimal risk of hypoglycemia, which is significantly lower than that associated with other usually prescribed antidiabetic medications such as insulin or sulfonylureas. Consequently, the use of SGLT2is helps prevent AF episodes that can be triggered by glycemic fluctuations in diabetic populations [72,73].

In addition, obesity is a well-known risk factor for AF development, as it contributes to structural and electrophysiological remodeling of the atria, increasing the likelihood of AF development and recurrence [1]. SGLT2is are recognized for their efficacy in reducing body weight among patients, particularly those with T2DM. The mechanism underlying this weight reduction involves the inhibition of glucose reabsorption in the renal proximal tubules, leading to osmotic diuresis and increased glycosuria. This glucose loss results in a caloric deficit, which subsequently contributes to a decrease in body weight [74]. Clinical trials and observational studies have consistently demonstrated that patients treated with gliflozins experience significant reductions in both body weight and adiposity, highlighting the potential of these agents not only for glycemic control but also for weight management [75]. Despite the moderate body weight reduction induced by gliflozins, the concurrent slight decrease in both systolic and diastolic BP and cardiac filling pressures can synergistically improve atrial substrate, contributing to their antiarrhythmic properties [76].

### 2.5. Regulation of Autonomic Nervous System Activity

The imbalance in the sympathetic and parasympathetic nervous systems’ tone of the heart is widely described as a crucial underlying mechanism in atrial arrhythmogenesis, contributing to the onset and recurrence of AF [77]. Peripheral sympatholytic effects—mediated by suppression of GPR41 activity—in aortic, cardiac, and renal tissues have been described in animal models after administration of dapagliflozin [78,79]. Also, SGLT2is possess a unique property of reducing interstitial fluid volume more significantly than intravascular fluid volume. This mechanism offers protective effects against neurohormonal activation triggered by changes in intravascular fluid volume [66]. Experimental studies have demonstrated evidence that the suppression of the hyperactive sympathetic tone can be achieved by moderating the adrenergic activity of the afferent sympathetic nerve, leading to a decreased activation of the renin–angiotensin–aldosterone system with possible beneficial effects on atrial electrophysiology [66].

### 2.6. Erythropoiesis and Hematocrit Levels

Additionally, the inhibition of SGLT2 confers further benefits by influencing erythropoiesis and hematocrit levels [3]. Research suggests that SGLT2is adjust medullary oxygen tension, which affects the function of myofibroblasts in the renal interstitium. This adjustment stimulates the production and release of erythropoietin, leading to a subsequent increase in hematocrit [3]. The rise in hematocrit not only enhances the systemic oxygen-carrying capacity but also significantly improves myocardial and renal bioenergetics [80]. Enhanced systemic tissue oxygen supply and metabolic capacity, combined with the pro-angiogenic and anti-inflammatory impact of erythropoiesis [80], is critical for maintaining optimal atrial function and may contribute to preserving atrial structure and function over time, thus preventing atrial arrhythmogenesis [81].

**Figure 1:**
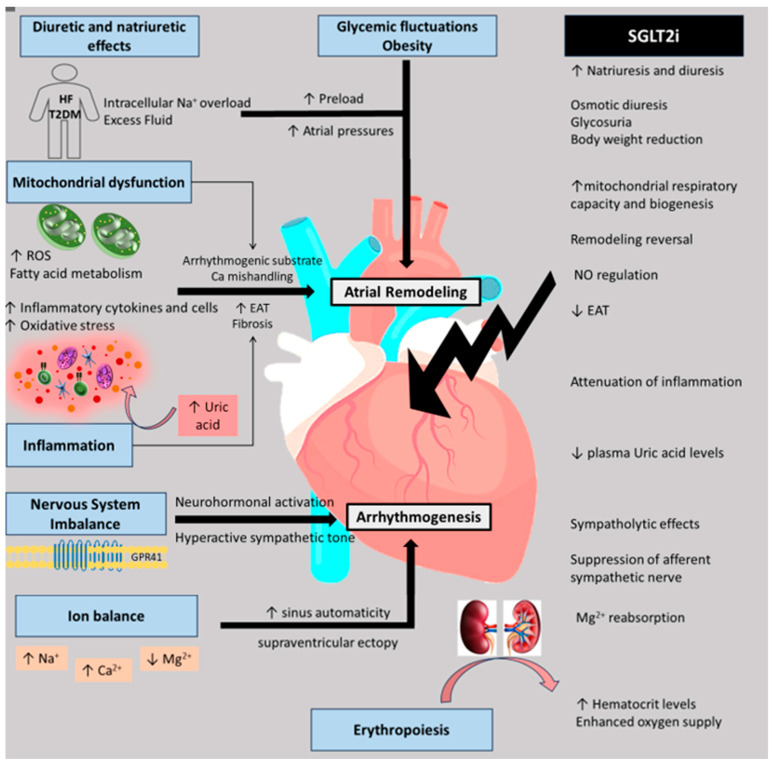
The potential underlying pathological mechanisms involved in the antiarrhythmic properties of SGLT2is.

In summary, SGLT2is show promise in intricately modifying the left atrial and pulmonary veins’ electrophysiological and anatomical substrate through direct and predominantly indirect mechanisms, encompassing structural and electrical remodeling, improved mitochondrial function and bioenergetics, reduced oxidative stress, systemic inflammation and induced cardiac fibrosis, restoration of ion balance, autonomic nervous system regulation, and ameliorated hemodynamics, thereby mitigating the initiation and progression of atrial arrhythmias including AF [19] (Figure 1).

## 3. Clinical Data

### 3.1. SGLT2is and AF Incidence

Many observational and randomized studies conducted across different countries and settings have examined the potential antiarrhythmic effects of SGLT2is on the incidence and prevalence of AF and/or atrial flutter (AFL) among various patient populations, primarily those with T2DM and secondarily with HF (Table 1).

#### 3.1.1. RCTs

Zelniker et al. conducted a post-hoc analysis of the DECLARE-TIMI 58 trial, finding that dapagliflozin compared to placebo reduced AF/AFL events by 19% (7.8 vs. 9.6 events per 1000 patient-years; HR 0.81, 95% CI: [0.68–0.95], *p* = 0.009) over 50.4 months [82]. Butt et al. analyzed data from the DAPA-HF trial, showing no significant reduction in new-onset AF with dapagliflozin compared to placebo (2.8 vs. 3.3 events per 100 person-years, respectively; HR 0.86, 95% CI: [0.60–1.22]) among patients with HFrEF over a median follow-up period of 18.2 months [83]. In addition, Li et al. conducted a pooled analysis from the CANVAS Program and CREDENCE trial and demonstrated that canagliflozin had no significant effect on AF/AFL events, though subgroup analysis suggested a possible benefit in patients without baseline atrial arrhythmia (HR 0.78, 95% CI: [0.62–0.99]) [84].

#### 3.1.2. Observational Studies

The majority of research investigating the potential antiarrhythmic properties of gliflozins is observational, predominantly retrospective. Specifically, Ling et al. in Taiwan observed a lower incidence of new-onset AF in a large cohort of T2DM patients treated with SGLT2is compared to DPP4is [85]. Similarly, Lee et al. identified a significantly reduced incidence of new-onset AF associated with SGLT2is compared to DPP4is over 67.6 months in an extensive Hong Kong cohort [86]. Additionally, Chan et al. conducted a nationwide Taiwanese study indicating a lower risk of new-onset AF with SGLT2is compared to both DPP4is and GLP-1RAs [87]. Similarly, in a U.S.-based study, Zhuo et al. demonstrated that SGLT2is were associated with reduced AF incidence among Medicare patients compared to DPP4is and GLP-1RAs [88]. A multicenter study in Scandinavia similarly found a lower adjusted incidence rate of new-onset AF with SGLT2is compared to GLP-1RAs [89]. Further, Lui et al. reported a lower risk of incident AF with SGLT2is compared to GLP-1RAs in another Hong Kong study [90]. Zhou et al., in a single-center Chinese study, documented a lower prevalence and risk of AF in HF patients treated with SGLT2is [91]. Lastly, Tanaka et al. observed fewer episodes of new-onset AF in patients with HF, particularly those with non-ischemic dilated cardiomyopathy, treated with SGLT2is over 6.1 years in a single-center study in Japan [92].

On the other hand, Persson et al. in a retrospective observational study in Scandinavia found no significant difference in new-onset AF incidence with dapagliflozin compared to DPP4is over 11.4 months [93]. Furthermore, Cesaro et al. in an international retrospective cohort study linked SGLT2is with a lower occurrence of new-onset cardiac arrhythmias, though the reduction in AF was not statistically significant [94].

In summary, many observational studies, predominantly retrospective, have consistently indicated a reduction in AF risk with SGLT2is, whereas RCTs presented mixed results, with some showing benefits and others not reaching statistical significance (Table 1). Nevertheless, it is highlighted that the observational studies may suffer from bias and confounding factors, despite propensity score matching, and RCTs, although more controlled, are fewer in number and their outcomes are heterogenous and conflicting. Interestingly, data about AF incidence was not a pre-defined endpoint of the studies and was typically reported as adverse events, thus with an inevitable risk of under-reporting. Hence, data collection may not have been conducted in a rigorous and systematic manner. Overall, the evidence indicates a promising antiarrhythmic benefit of SGLT2is in reducing the risk of new-onset AF, especially in observational settings. However, the heterogeneity in study outcomes, population characteristics, duration of follow-up, and specific SGLT2is used, as far as the potential biases, necessitate further more extensive and rigorous RCTs to establish definitive conclusions and understand the mechanisms involved.

**Table 1:** Studies investigating the potential antiarrhythmic effects of SGLT2is on AF incidence.

Study ID	Setting (Country)	Type of Study	Population	SGLT2is	Follow-Up	Main Outcomes
Zelniker et al., 2020 [82]	882 participating institutions in 33 countries	RCT (post-hoc analysis)	Patients with **T2DM** and either multiple risk factors for atherosclerotic CVD (n = 10,186) or known atherosclerotic CVD (n = 6974) in the DECLARE-TIMI 58 trial, irrespectively of baseline prevalence of AF/AFL.	DAPA	50.4 months	DAPA reduced the risk of **AF/AFL events** by 19% (7.8 vs. 9.6 events per 1000 patient-years; HR 0.81, 95% CI: [0.68–0.95], *p* = 0.009). There was no effect modification by sex, history of ischemic stroke, HbA1c, BMI, BP, or eGFR (all *p* for interaction > 0.20). DAPA also reduced the total number (first and recurrent) of **AF/AFL events** (337 vs. 432; incidence rate ratio 0.77, 95% CI: [0.64–0.92], *p* = 0.005).
Butt et al., 2022 [83]	410 participating institutions in 20 countries	RCT (post-hoc analysis)	Patients with **HFrEF** (LVEF ≤ 40%) and without AF (history of AF or AF on enrolment ECG) in the DAPA-HF trial (n = 2834).	DAPA	18.2 months	Among patients without AF at baseline, DAPA did not significantly decrease the risk of **new-onset AF** compared with placebo (2.8 vs. 3.3 events per 100 person-years respectively; HR 0.86, 95% CI: [0.60–1.22]).
Li et al., 2022 [84]	667 participating institutions in 30 countries	RCT (post-hoc pooled analysis)	Participants with **T2DM** and high risk of CVD or CKD were included and randomly assigned to CANA or placebo. Pooled analysis from CANVAS Program and CREDENCE trial.	CANA	47 months	CANA had no detectable effect on **AF/AFL events** (HR 0.82, 95% CI: [0.67–1.02]) compared with placebo. Subgroup analysis, however, suggested a possible reduction in AF/AFL in those with no AF/AFL history at baseline (HR 0.78, 95% CI: [0.62–0.99]). Results were similar after adjusting for age, gender, BMI, HbA1c, and eGFR.
Persson et al., 2018 [93]	Denmark, Norway, and Sweden	Observational Multinational Retrospective Study (CVD-REAL Nordic)	Patients with **T2DM** (n = 10,227 new users of DAPA and n = 30,681 new users of DPP4i) matched 1:3 by PS.	DAPA	11.4 months	DAPA was not associated with a lower risk of **new-onset AF incidence** compared to DPP4is (1.47/100 patient-years vs. 1.58/100 patient-years, respectively, HR 0.92, 95% CI: [0.76–1.12]).
Ling et al., 2020 [85]	Chang Gung Memorial Hospital (Taiwan)	Observational Retrospective Single-center Study	Patients with **T2DM** and without AF at baseline (n = 15,606 and n = 12,383 treated with SGLT2is and DPP4i, respectively). PS weighting was used to balance covariates across study groups.	EMPA DAPA CANA	17.76 and 12.6 months for SGLT2i and DPP4i groups, respectively	The use of SGLT2is was associated with a lower risk of **new-onset AF** compared with DPP4i (HR 0.61, 95% CI: [0.50–0.73], *p* < 0.001). Subgroup analysis revealed similar antiarrhythmic results across several subgroups including old age, females, the presence of CVD, HBA1c, and CKD.
Tanaka et al., 2021 [92]	Kobe University Hospital of Japan	Observational Retrospective Single-center Study	Patients with non-ischemic DCM (n = 218) without AF at baseline and **LVEF < 45%**.	EMPA DAPA CANA	6.1 years	Of the 60 patients with T2DM, the SGLT2i users (32 patients (53.3%)) experienced fewer episodes of **new-onset AF** compared to non-SGLT2i users (log-rank *p* = 0.04).
Zhou et al., 2022 [91]	First Affiliated Hospital of Anhui Medical University (China)	Observational Retrospective Single-center Study	Patients with **HF** (n = 903), including 78 participants with AF and 825 participants without AF at baseline.	EMPA DAPA CANA	N/A	SGLT2i users experienced a lower **prevalence of AF** (8.4% vs. 12.1%, *p* < 0.001) and a lower risk of developing AF (OR 0.76, 95% CI: [0.70–0.85], *p* < 0.001) compared to non-SGLT2i users. The effect of SGLT2is on AF episodes was consistent irrespectively of age, gender, BMI, and eGFR.
Lee et al., 2022 [86]	Hong Kong (China)	Observational Retrospective Population-based Cohort Study	Patients with **T2DM** (n = 61,233) treated with SGLT2is (n = 21,713) or DPP4is (n = 39,510). PS matching (1:1 ratio) was performed.	EMPA DAPA CANA ERTU	67.6 months	Patients treated with SGLT2is demonstrated lower incidence of **new-onset AF** (1.96% vs. 2.78%, SMD = 0.05) compared to DPP4i users. Cox regression found that SGLT2i users showed lower risk of **new-onset AF** (HR 0.68, 95% CI: [0.56–0.83], *p* = 0.0001) after adjusting for significant confounder factors.
Chan et al., 2022 [87]	Taiwan	Observational Nationwide Retrospective Cohort Study	Patients with **T2DM** without preexisting AF receiving GLP-1RA (n = 344,893), SGLT2i (n = 44,370), and DPP4i (n = 393,100) were enrolled. A 1:1 PS matching to balance covariates across paired study groups was used.	EMPA DAPA CANA ERTU	2.03 and 2.01 years for the paired SGLT2i and DPP4i groups, and 2.24 and 2.24 years for the SGLT2i and GLP-1RA groups, respectively	SGLT2i administration was associated with lower risk of **new-onset AF** compared with either DPP4i (HR 0.90, 95% CI: [0.84–0.96], *p* = 0.0028) or GLP-1RA (HR 0.74; 95% CI: [0.63–0.88], *p* = 0.0007) treatment after PSM. After subgroup analysis, DAPA was associated with a lower risk of **new-onset AF** compared with DPP4i (*p* interaction = 0.02)
Zhuo et al., 2022 [88]	USA	Observational Nationwide Retrospective Cohort Study	Patients (aged ≥66 years) with **T2DM** and without known history of AF who were enrolled in Medicare fee-for-service. New users of SGLT2is were 1:1 PS-matched to new users of a DPP-4i (n = 74,868) or GLP-1RA (n = 80,475).	EMPA DAPA CANA	191 and 214 days among SGLT2i and DPP4i users, respectively, and 188 and 173 days among SGLT2i and GLP-1RA users, respectively	The risk of **incident AF** was lower in the SGLT2i users than the matched DPP4i arm (HR 0.82, 95% CI: [0.76–0.89]; RD–3.7, 95% CI: [−5.2 to −2.2] per 1000 person-years) or the matched GLP-1RA group (HR 0.90, 95% CI: [0.83–0.98], RD −1.8, 95% CI: [−3.2 to −0.3] per 1000 person-years).
Hsiao et al., 2022 [95]	Taiwan	Observational Multicenter Retrospective Cohort Study	Patients with **T2DM** treated either with SGLT2is (n = 16,566) or GLP-1RAs (n = 2746). PS weighting was used to balance the baseline covariates.	EMPA DAPA CANA	1.52 ± 0.74 years for the SGLT2i users and 1.33 ± 1.12 years for the GLP-1RA group	SGLT2i users experienced a significantly lower risk of **new-onset AF** compared with GLP-1RA users (sub-distribution HR 0.72, 95% CI: [0.54–0.97], *p* = 0.028). Subgroup analysis revealed similar findings among different high-risk subgroups like older patients, female patients, and patients with CVD or CKD.
Jhuo et al., 2022 [96]	Kaohsiung Medical University Hospital of Taiwan	Observational, Retrospective, Single-Center Study	Patients with **T2DM** (n = 9609) without a known history of arrhythmia and antiarrhythmic medication who were prescribed SGLT2i (n = 3203) vs. non-SGLT2i users (n = 6406).	N/A	51.50 ± 4.23 months	Multivariate analysis showed that SGLT2i administration was associated with significantly lower incidence of **new-onset AF** (HR 0.56, 95% CI: [0.35–0.88], *p* = 0.013) than for non-SGLT2i users.
Cesaro et al., 2022 [94]	Italy Belgium Bulgaria	Observational Multicenter International Retrospective Cohort Study	Patients with **T2DM and AMI** (n = 646) from the SGLT2i AMI PROTECT registry (NCT05261867). SGLT2i users (n = 111) before AMI were compared to non-SGLT2i users (n = 535).	N/A	5 days (hospital stay)	In the multivariate logistic regression model, SGLT2i administration was associated with the lower **occurrence of NOCAs** (OR 0.35, 95% CI: [0.14–0.86], *p* = 0.022), but it was not an independent predictor of **AF occurrence**, which showed a reduction, without reaching statistical significance (OR 0.40, 95% CI: [0.14–1.14], *p* = 0.086).
Engström et al., 2023 [89]	Denmark Norway Sweden	Observational Multicenter International Retrospective Cohort Study	Patients with **T2DM** without a history of AF who were newly prescribed an SGLT2i (n = 79,343) or an GLP-1RA (n = 57,613) adjusted for baseline covariates with PS weighting.	EMPA DAPA CANA ERTU	N/A	The adjusted incidence rate of **new-onset AF** was 8.6 per 1000 person-years for new SGLT2i users compared with 10.0 per 1000 person-years for new GLP-1RA users (adjusted HR 0.89, 95% CI: [0.81–0.96]). No statistically significant heterogeneity of the adjusted HRs was observed between subgroups of patients with and without a history of HF or CVD.
Fawzy et al., 2023 [97]	More than 60 centers including in TriNetX global research network across seven countries (mainly in USA)	Observational Retrospective Cohort Study	Patients with **T2DM** who were treated either with (n = 131,189) or without SGLT2is (n = 2,692,985). After PSM, 131,188 patients remained in each group.	N/A	24 months	Patients who were treated with SGLT2is were experienced a significantly lower risk of **incident AF** compared to non-SGLT2i users (HR 0.81, 95% CI: [0.76–0.84]).
Lui et al., 2023 [90]	Hong Kong Hospital Authority	Observational, Population-based, Retrospective Cohort Study	5840 patients with **T2DM** (2920 SGLT2i users; 2920 GLP-1RA users) were matched one to one by PS.	EMPA DAPA CANA	17 months	SGLT2i administration was associated with lower risk of **incident AF** (HR 0.43, 95% CI: [0.23–0.79], *p* = 0.006) compared to GLP-1RA users.
Eroglu et al., 2024 [98]	UK	Observational, Population-based, Retrospective Cohort Study	Patients with **T2DM** who initiated a new antidiabetic drug class without a diagnosis of AF or AFL (n = 142,447).	N/A	N/A	SGLT2is were associated with a statistically significant decreased risk of AF compared to other hypoglycemic agents (aHR 0.77, 95% CI: [0.68–0.88])
Li et al., 2024 [99]	USA	Observational Retrospective Cohort Study	Older patients with **T2DM** and no history of AF (n = 97,436) who were prescribed either SGLT2is or DPP4is.	EMPA DAPA CANA	361 days	SGLT2is were associated with a significantly lower risk of **incident AF** (HR 0.73, 95% CI: [0.57–0.91], *p* = 0.01) than DPP4is. Non-Hispanic white individuals and patients with existing CVD or CKD experienced significantly lower risk in subgroup analysis.

AF: Atrial Fibrillation, AFL: Atrial Flutter, AMI: Acute Myocardial Infarction, BMI: Body Mass Index, BP: Blood Pressure, CANA: Canagliflozin, CI: Confidence Interval, CKD: Chronic Kidney Disease, CREDENCE: Canagliflozin and Renal Events in Diabetes with Established Nephropathy Clinical Evaluation, CVD: Cardiovascular Disease, DAPA: Dapagliflozin, DAPA-HF: Dapagliflozin And Prevention of Adverse-outcomes in Heart Failure trial, DECLARE-TIMI 58: Dapagliflozin Effect on Cardiovascular Events-Thrombolysis in Myocardial Infarction 58, DCM: Dilated Cardiomyopathy, DPP4is: Dipeptidyl peptidase 4 inhibitors, eGFR: estimated Glomerular Filtration Rate, EMPA: Empagliflozin, ERTU: Ertugliflozin, GLP-1: Glucagon-Like Peptide 1, GLP-1RAs: GLP-1 receptor agonists, HF: Heart Failure, HFrEF: Heart Failure with Reduced Ejection Fraction, HR: Hazard Ratio, LVEF: Left Ventricular Ejection Fraction, NOCA: New-Onset Cardiac Arrhythmias, N/A: Not Applicable, OR: Odds Ratio, PS: Propensity Score, RCT: Randomized Clinical Trial, SGLT2is: Sodium-Glucose cotransporter 2 inhibitors, SMD: Standardized Mean Difference, T2DM: Type 2 Diabetes Mellitus, UK: United Kingdom, USA: United States of America.

### 3.2. SGLT2is and AF Recurrence

Many observational and randomized studies conducted across different countries and settings have examined the potential antiarrhythmic effects of SGLT2is on the recurrence of AF among various patient populations, primarily those with T2DM undergoing catheter ablation (Table 2). SGLT2is may have a substantial impact on the maintenance of sinus rhythm in patients with AF [100]. However, existing evidence from both preclinical and clinical studies is currently inconclusive. As a result, SGLT2is are not yet endorsed as a standard pharmacological intervention for reducing the recurrences of AF [101].

#### 3.2.1. RCTs

Kishima et al. conducted a randomized clinical trial at Hyogo College of Medicine in Japan involving 70 AF patients with T2DM post-ablation. Patients were randomized 1:1 to either the tofogliflozin or anagliptin group. The study found that the AF recurrence rate was significantly higher in the anagliptin arm compared to the SGLT2is arm (47% vs. 24%, *p* = 0.0417) [102].

#### 3.2.2. Observational Studies

In the observational retrospective cohort study conducted by Haloot et al. (2021) across 48 healthcare organizations, primarily in the United States, after propensity score matching it was demonstrated that the administration of SGLT2is was associated with a significantly decreased risk of cardioversion (HR 0.921, 95% CI: [0.841–0.999], *p* = 0.0245) [103]. In addition, Luo et al. (2022) carried out an observational retrospective cohort study in China involving patients with T2DM and AF who underwent ablation. The study compared 79 patients in the dapagliflozin group to 247 in the control group over a mean follow-up of 15.5 ± 8.9 months. The SGLT2i group exhibited a lower AF recurrence rate (27.8% vs. 44.9%, *p* = 0.007), and treatment with dapagliflozin was associated with a reduced risk of atrial arrhythmia recurrence (HR 0.614, 95% CI: [0.387–0.974], *p* = 0.038) [104]. Similarly, according to the observational retrospective study by Abu-Qaoud et al. using the TriNetX research network and involving more than 4450 patients with T2DM post-catheter ablation for AF, SGLT2i users experienced significantly lower risks of cardioversion, new antiarrhythmic drug therapy, and redo AF ablation compared to controls (aOR 0.68, 95% CI: [0.602–0.776], *p* < 0.0001) [100].

Liu et al. conducted an observational retrospective single-center study at Chang Gung Memorial Hospital in Taiwan, involving 122 AF patients with T2DM undergoing catheter ablation. The study observed that patients treated with SGLT2is had a significantly higher rate of sinus rhythm maintenance (92.5% vs. 72.1%, *p* = 0.015), and SGLT2i use was independently associated with reduced AF recurrence (HR 0.18, 95% CI: [0.04–0.79], *p* = 0.023) [105]. Moreover, Zhao et al. performed an observational retrospective multicenter study in China, including 525 T2DM patients with AF undergoing initial catheter ablation. SGLT2i users (n = 138) were matched with non-users (n = 387) in a 1:3 ratio. Over an 18-month follow-up, SGLT2i users had a significantly lower AF recurrence rate compared to non-users (HR 0.63, 95% CI: [0.44–0.90], *p* = 0.007) [106]. Finally, Qi et al. conducted a single-center observational retrospective study at Beijing Chaoyang Hospital, China, involving 182 T2DM patients with persistent AF undergoing their first radiofrequency ablation. Over a mean follow-up of 16.2 months, SGLT2i users showed a significantly lower risk of AF recurrence compared to non-users (adjusted HR 0.65, 95% CI: [0.28–0.83], *p* < 0.01) [107].

**Table 2:** Studies investigating the potential antiarrhythmic effects of SGLT2is on AF recurrence.

Study ID	Setting (Country)	Type of Study	Population	SGLT2i	Follow-Up	Outcomes
Haloot et al., 2021 [103]	48 Healthcare Organizations including in TriNetX global research network (mainly in the United States)	Observational Retrospective Cohort Study	**AF** patients on a SGLT2i (n = 26,294) and not on a SGLT2i (n = 1,368,518). After propensity score matching, 26,269 patients in each cohort.	EMPA DAPA CANA	N/A	SGLT2i administration was associated with significantly decreased risk of **cardioversion** (HR 0.921, 95% CI: [0.841–0.999], *p* = 0.0245).
Kishima et al., 2022 [102]	Hyogo College of Medicine, Nishinomiya (Japan)	RCT	AF patients with **T2DM** (n = 70) after **AF ablation**. Randomization 1:1 (tofogliflozin vs. anagliptin group).	TOFO	N/A	**AF recurrence rate** was higher in the anagliptin arm compared to the TOFO arm (15 of 32 patients [47%] vs. 9 of 38 patients [24%], *p* = 0.0417).
Luo et al., 2022 [104]	First Affiliated Hospital of Zhengzhou University (China)	Observational Retrospective Cohort Study	Patients with **T2DM** and AF, who underwent **AF ablation**. SGLT2is group (n = 79) and the control group (n = 247).	DAPA	15.5 ± 8.9 months	The DAPA group had a lower **AF recurrence rate** than the control group (27.8% vs. 44.9%, *p* = 0.007). Treatment with DAPA was associated with a lower risk of **recurrence of atrial arrhythmias** (HR 0.614, 95% CI: [0.387–0.974], *p* = 0.038) in multivariable Cox regression models.
Abu-Qaoud et al., 2023 [100]	TriNetX research network	Observational Retrospective Cohort Study	Patients with **T2DM** after **CA for AF** were divided by PSM into SGLT2i users (n = 2225) and non-SGLT2i users (n = 2225).	N/A	12 months	Patients receiving gliflozins were experienced a significantly lower risk of **cardioversion, new AAD therapy, and re-do AF ablation** compared to controls (aOR 0.68, 95% CI: [0.602–0.776], *p* < 0.0001).
Liu et al., 2023 [105]	Chang Gung Memorial Hospital (Taiwan)	Observational, Retrospective, Single-Center Study	Patients with AF and **T2DM** (n = 122) undergoing **CA**.	EMPA DAPA CANA	12 months	The **maintenance of sinus rhythm rate** was significantly higher in the SGLT2i-treated patients compared to controls (92.5% vs. 72.1%, respectively, *p* = 0.015), and SGLT2i administration was independently associated with **AF recurrence** after CA (HR 0.18, 95% CI: [0.04–0.79], *p* = 0.023).
Zhao et al., 2023 [106]	China	Observational, Retrospective, Multicenter Study	Patients with **T2DM** and **AF** undergoing **initial CA** were included. SGLT2i users (n = 138) were matched by PSM with non-SGLT2i users (n = 387) in a 1:3 ratio.	N/A	18 months	Patients receiving SGLT2is experienced a significantly lower **AF recurrence** rate compared with the non-SGLT2i users (HR 0.63, 95% CI: [0.44–0.90], *p* = 0.007).
Qi et al., 2024 [107]	Beijing Chaoyang Hospital (China)	Observational, Retrospective, Single-Center Study	Patients with **T2DM** and **persistent AF** (n = 182), undergoing their first **radiofrequency ablation**.	EMPA DAPA	16.2 months	SGLT2i users experienced a significantly lower risk of **AF recurrence** compared to non-SGLT2i users (adjusted HR 0.65, 95% CI: [0.28–0.83], *p* < 0.01).
Noh et al., 2024 [108]	Veterans’ Health Service Medical Center (Korea)	Observational, Retrospective, Single-Center Study	Patients who underwent **AF CA** (n = 272), including non-SGLT2i users (n = 199) and DAPA users (n = 73).	DAPA	18 months	SGLT2i administration was associated with a significantly decreased risk of **AF recurrence** (aHR 0.15, 95% CI: [0.07–0.32], *p* < 0.001). DAPA users experienced a significantly higher period without total arrhythmia recurrence compared to non-DAPA users (log-rank test *p* < 0.01)
Fichadiya et al., 2024 [109]	Alberta (Canada)	Observational, Retrospective Population-based Cohort Study	Patients with **T2DM and AF** (n = 2242) were included. Adults prescribed SGLT2is were matched 1:1 to those prescribed DPP4is based on time-conditional PS.	CANA DAPA EMPA	36 months	The primary endpoint (a **composite of AF-related healthcare utilization,** i.e., hospitalization, ED visits, electrical cardioversion, or CA) occurred in 8.7% (n = 97) of the SGLT2i users compared to 10.0% (n = 112) of DPP4i users (aHR 0.73, 95% CI: [0.55–0.96], *p* = 0.03).

AAD: Antiarrhythmic Drug, AF: Atrial Fibrillation, CA: Catheter Ablation, CANA: Canagliflozin, CI: Confidence Interval, DAPA: Dapagliflozin, DPP4is: Dipeptidyl peptidase 4 inhibitors, ED: Emergency Department, EMPA: Empagliflozin, HR: Hazard Ratio, N/A: Not Applicable, OR: Odds Ratio, RCT: Randomized Clinical Trial, SGLT2is: Sodium-Glucose cotransporter 2 inhibitors, T2DM: Type 2 Diabetes Mellitus, TOFO: Tofogliflozin.

#### 3.2.3. Meta-Analyses

According to a recent meta-analysis addressing the effect of SGLT2is on sinus rhythm maintenance in patients with a history of AF ablation, which included four randomized and observational studies [100,102,104,105] and more than 4900 patients, all with concomitant T2DM, it was found that patients receiving gliflozins had significantly lower odds of AF recurrence compared to non-SGLT2i users (OR = 0.60; 95% CI: [0.45–0.79], *p* < 0.001) [110]. Similar were the findings of a recently published meta-analysis of four studies in which SGLT2is were associated with lower AF recurrence in comparison to the control group (OR 0.61, 95% CI: [0.54–0.69]) after 12 months of follow-up [106].

In summary, the current clinical data suggest a promising role for SGLT2is in reducing AF recurrence, particularly in patients with concurrent T2DM. The studies consistently report a favorable impact of SGLT2is on maintaining sinus rhythm and decreasing the need for cardioversion and additional antiarrhythmic therapies. However, the variability in study designs, populations, SGLT2i types, and follow-up durations necessitates cautious interpretation. Observational studies, while insightful, have inherent limitations such as potential biases and confounding factors that may affect the outcomes. The RCT by Kishima et al. [102] provides more robust evidence supporting the antiarrhythmic potential of SGLT2is, yet larger and more diverse clinical trials are needed to confirm these findings and establish comprehensive guidelines. In conclusion, while the current evidence is promising, the heterogeneity and limitations of existing studies mean that SGLT2is cannot yet be recommended as a standard treatment for AF recurrence. Future research, including well-designed RCTs and mechanistic studies, is essential to validate the prospective antiarrhythmic properties of SGLT2is and to ascertain their potential routine application in patients with AF, irrespective of their baseline diabetes or HF status.

## 4. Future Directions

Future research should focus on conducting large-scale RCTs with pre-specified AF endpoints to definitively establish the efficacy of SGLT2is in reducing AF incidence and progression. Studies should aim to standardize population characteristics, follow-up durations, and specific SGLT2i agents to address current heterogeneities. Notably, there is no specific RCT primarily looking at SGLT2is and AF in HF patients and all data are from post hoc analyses only, so future studies are needed towards this direction. Additionally, mechanistic investigations are needed to elucidate the principal pathways through which gliflozins exert their antiarrhythmic effects, especially in AF populations. Understanding these mechanisms will not only clarify the role of SGLT2is in AF management but also enhance the development of targeted therapeutic strategies for patients with coexisting comorbidities including HF, T2DM, and CAD.

Several trials are ongoing including the EMPA-AF (NCT04583813) with empagliflozin in patients with T2DM or obesity, HF, and AF, the DAPA-AF with dapagliflozin in patients undergoing AF catheter ablation, the BEYOND trial (Clinical BEnefit of sodium–glucose cotransporter-2 inhibitors in rhYthm cONtrol of atrial fibrillation in patients with diabetes mellitus; NCT05029115) aiming to enroll patients with AF and T2DM and investigate the recurrence of AF at 12 months among SGLT2i users and non-SGLT2i users [111], and the DETAIL-CMIV study, which will determine whether dapagliflozin, added to guideline-recommended post-operative AF therapies, safely reduces the recurrence rate of AF in patients with and without T2DM or HF [112]. Finally, the SUPRESS-AF study, a single-center observational study, aims to investigate the impact of SGLT2is (dapagliflozin and empagliflozin) on AF recurrence rate in patients with paroxysmal or persistent AF and HF with preserved or mildly reduced ejection fraction using objective means (smartwatches).

## 5. Conclusions

In conclusion, this comprehensive review highlights the potential role of SGLT2is in the management of atrial fibrillation. While observational studies indicate a promising reduction in AF risk associated with SGLT2i use, randomized controlled trials present inconsistent results. The heterogeneity in study designs, patient populations, and follow-up durations necessitates further rigorous research to confirm these findings and elucidate the mechanisms by which SGLT2is may confer antiarrhythmic benefits. Future studies should aim to provide more definitive evidence to guide clinical practice in the prevention and management of AF in patients with comorbid conditions such as HF and T2DM.

## Data Availability

Our study data are available from the corresponding study author (N.F.) upon reasonable request.
